# Oceanic nutrient rise and the late Miocene inception of Pacific oxygen-deficient zones

**DOI:** 10.1073/pnas.2204986119

**Published:** 2022-11-02

**Authors:** Xingchen Tony Wang, Yuwei Wang, Alexandra Auderset, Daniel M. Sigman, Haojia Ren, Alfredo Martínez-García, Gerald H. Haug, Zhan Su, Yi Ge Zhang, Birger Rasmussen, Alex L. Sessions, Woodward W. Fischer

**Affiliations:** ^a^Department of Earth and Environmental Sciences, Boston College, Chestnut Hill, MA 02467;; ^b^Department of Climate Geochemistry, Max Planck Institute for Chemistry, 55128 Mainz, Germany;; ^c^Department of Geosciences, Princeton University, Princeton, NJ 08544;; ^d^Department of Geosciences, National Taiwan University, Taipei 106, Taiwan;; ^e^Department of Earth Sciences, ETH Zürich, 8092 Zürich, Switzerland;; ^f^Department of Physics, University of Toronto, Toronto, ON M5S 1A7, Canada;; ^g^Department of Oceanography, Texas A&M University, College Station, TX 77843;; ^h^School of Earth Sciences, The University of Western Australia, Crawley, WA 6009, Australia;; ^i^Division of Geological and Planetary Sciences, California Institute of Technology, Pasadena, CA 91101

**Keywords:** ocean deoxygenation, nutrient cycling, nitrogen isotopes, late Miocene

## Abstract

The modern Pacific Ocean hosts the largest oxygen-deficient zones (ODZs), where oxygen concentrations are so low that nitrate is used to respire organic matter. The history of the ODZs may offer key insights into ocean deoxygenation under future global warming. In a 12-My record from the southeastern Pacific, we observe a >10‰ increase in foraminifera-bound nitrogen isotopes (^15^N/^14^N) since the late Miocene (8 to 9 Mya), indicating large ODZs expansion. Coinciding with this change, we find a major increase in the nutrient content of the ocean, reconstructed from phosphorus and iron measurements of hydrothermal sediments at the same site. Whereas global warming studies cast seawater oxygen concentrations as mainly dependent on climate and ocean circulation, our findings indicate that modern ODZs are underpinned by historically high concentrations of seawater phosphate.

## Significance

Oxygen is critical to marine ecosystems. The ocean contains oxygen-deficient zones (ODZs) that have been expanding over the past several decades. Predicting future changes in ocean deoxygenation is important for marine ecosystems and human societies that rely on fisheries. Understanding the history of the ocean's oxygen content can provide important insights into how ODZs will behave in the future with climate change and increasing anthropogenic nutrient inputs. In this study, we found that the largest ODZs on our planet were much smaller eight million years ago than they are today. Furthermore, our observations revealed that the expansion of the ODZs was mainly driven by increasing oceanic nutrient content and primary productivity. This finding provides valuable context for ocean deoxygenation processes in the modern and future ocean.

The oxygen (O_2_) content of the ocean is critical for the health of marine ecosystems and productive fisheries. Observations show that the oceans have been losing O_2_ over the past five decades in both open ocean and coastal zones, attributed in part to anthropogenic climate change and coastal nutrient discharge (1, 2). However, simulating the lowest O_2_ regions of the ocean, the oxygen-deficient zones (ODZs), is a challenge for climate models, undercutting the predictions of future ocean deoxygenation (3).

In general, the O_2_ content in the ocean interior is determined by the balance between O_2_ supply through ocean circulation and O_2_ consumption by the respiration of sinking organic matter imported from the surface ocean. In the ODZs, ocean circulation patterns minimize O_2_ supply (4), and wind-driven upwelling drives high productivity in overlying surface waters and thus a rain of sinking organic matter, causing O_2_ concentrations to fall below ∼5 μmol/L, at which point nitrate is used for microbial respiration (5). Three major ODZs are found in the modern ocean, two of which are located in the eastern Pacific Ocean (the Eastern Tropical South Pacific [ETSP] and the Eastern Tropical North Pacific Ocean) at depths of roughly 150 to 500 m (*SI Appendix*, Fig. S1). Observations have demonstrated the expansion of the Pacific ODZs over the past several decades (6). However, it remains unclear whether they will continue to expand under global warming, because the O_2_ supply to the depths of the ODZs as well as the productivity in overlying surface waters may be affected by regional changes in atmospheric and oceanic conditions (3, 7). For example, some studies have suggested that, on longer timescales (i.e., more than a century), the ODZs may shrink after the initial expansion (8).

The history of the ODZs may provide important insights into their sensitivities to global warming and other human perturbations. However, reconstructions of variations in Pacific ODZs have thus far been restricted mainly to the Pleistocene (9, 10), with little known on longer timescales such as during past warm periods (e.g., the Pliocene, the Miocene). We measured foraminifera-bound δ^15^N (where δ^15^N = [(^15^N/^14^N_sample_)/(^15^N/^14^N_air_) − 1] × 1,000) to reconstruct a 12-Mya history of the South Pacific ODZs, which reveals a large, gradual expansion since the late Miocene. We also observe a concomitant rise in phosphate concentrations ([PO_4_^3−^]) in the South Pacific reconstructed over the same interval, using the phosphorus (P) and iron (Fe) content of hydrothermal sediments. Taken together, these data illuminate nutrient dynamics and their impacts on the O_2_ content of the ocean, the global carbon cycle, and climate change since the late Miocene.

**Fig. 1.**
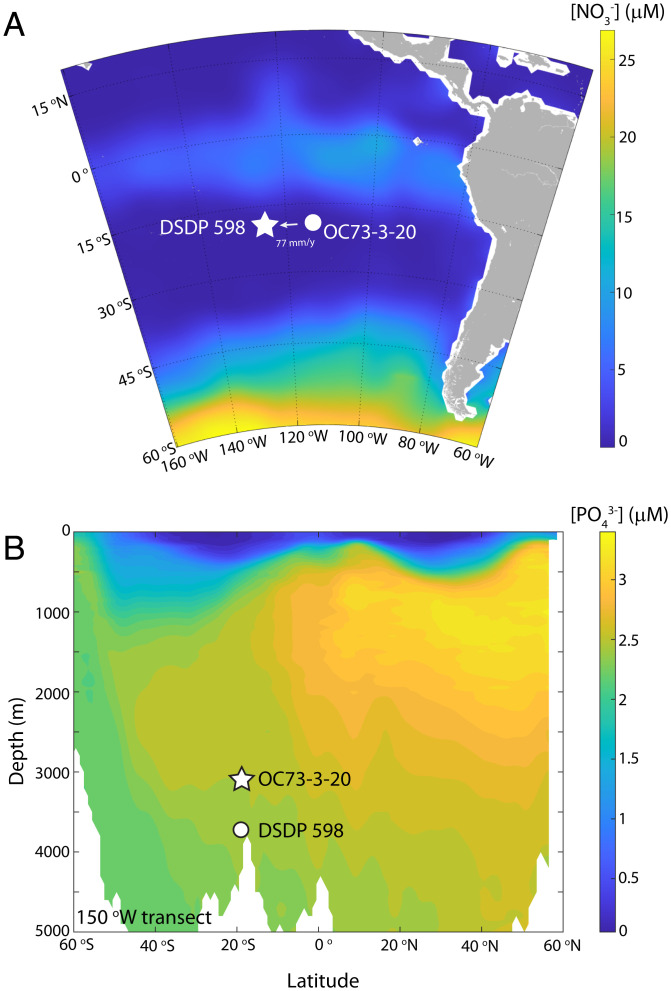
Modern nutrient distributions at the study sites. Maps show annual mean surface nitrate concentration (*A*) and transect of phosphate concentration in the Pacific Ocean along 150° W (*B*). OC-73–3-20 is currently 8 km west of the SEPR axis. With an estimated spreading rate of 77 mm/y, DSDP 598 would have been close to the current location of OC-73–3-20 12 Mya. Nitrate and phosphate concentration data are from *World Ocean Atlas*. To show the entire range of phosphate concentrations in the Pacific Ocean, 150° W data were used in *B* instead of 120° W.

Our study sites Deep Sea Drilling Project (DSDP) 598 (19.00 ^o^S, 124.68 ^o^W, 3,699 m) and OC-73–3-20 (19.25 °S, 113.58 °W, 3,081 m) are on the Southern Eastern Pacific Rise (SEPR; Fig. 1), a midocean ridge system with fast spreading rate and high hydrothermal activity (11). Today, OC-73–3-20 lies ∼8 km west of the SEPR axis, with DSDP 598 located ∼1,150 km to the west of OC-73–3-20. With an estimated spreading rate of 77 mm/y for the SEPR (12), DSDP 598 would have been only ∼200 km away from the SEPR axis 12 Mya. In this study, the 12-My records were generated from DSDP 598. Core OC-73–3-20, which is too short for long-term reconstructions, was used as a late Pleistocene comparison site to validate the proxies and correct for paleodepth variations.

These SEPR sites are ideal for the reconstruction of the history of Pacific ODZs for two reasons. First, these sites are close to the ETSP and the thermocline is directly affected by the ODZs through the transport of the South Equatorial Current, resulting in a high-nitrate δ^15^N of 12 to 13‰ from the water column denitrification in the ODZs (*SI Appendix*, Fig. S1) (13). Second, these sites are located within the oligotrophic zone of the South Pacific gyre, with complete nitrate consumption by phytoplankton in the surface ocean. The foraminifera-bound δ^15^N records from the SEPR sites, unlike the records from within the ODZs, are not complicated by fractionations due to incomplete nitrate consumption. Previous studies suggest that in ocean regions with complete nitrate consumption, foraminifera-bound δ^15^N is a proxy for the thermocline nitrate δ^15^N, because the thermocline nitrate is the dominant source of N to the euphotic zone, even in regions with relatively high rates of N_2_ fixation (14). Hence, it is expected that the foraminifera-bound δ^15^N from the SEPR provides a direct measure of its thermocline nitrate δ^15^N and thus the history of ODZs in the ETSP. Indeed, we observed very high Holocene-age δ^15^N values (14 to 16‰) in OC-73–3-20 (*SI Appendix*, Fig. S2), consistent with the high δ^15^N values seen in the modern thermocline nitrate. The Pleistocene foraminifera-bound δ^15^N in DSDP 598 matches the Holocene δ^15^N values observed in OC-73–3-20, supporting the use of DSDP 598 foraminifera-bound δ^15^N to reconstruct the long-term history of the ODZs in the ETSP.

In addition to the foraminifera-bound δ^15^N record, the SEPR sites provide a unique opportunity to reconstruct the nutrient content in the deep Pacific Ocean using the P and Fe content in these hydrothermal sediments. Phosphate removal by Fe oxyhydroxides in midocean ridges has long been recognized as a sink of P from seawater (15). The fast adsorption/scavenging of phosphate by abundant Fe oxyhydroxides is limited by the available phosphate, such that the Fe:P ratio in hydrothermal sediments is mainly influenced by the ambient deep-ocean [PO_4_^3−^]. It has been observed that the Fe:P ratio in hydrothermal precipitates displays a tight correlation with the in situ [PO_4_^3−^] (16). Furthermore, the hydrothermal phosphate sink has a minimal impact on the local phosphate concentration (i.e., ≤0.1 μM) (17), such that the processes that generate the Fe-P tracer do not self-limit the P scavenging process. These mechanics provided an approach to use measurements of the Fe and P contents of hydrothermal sediments as a proxy for seawater [PO_4_^3−^] in the past.

Although the mechanics of the Fe-bound P proxy are well understood, this proxy has been little used in paleoceanographic reconstructions, mainly because of a lack of suitable sedimentary materials. The SEPR sites provide a unique opportunity to apply this proxy because (1) the sites are adjacent to an active hydrothermal vent with high Fe oxyhydroxides production; (2); Fe-bound P is the dominant P species in the sediments; (3) the sites are in an oligotrophic setting with very low organic matter content, minimizing postdepositional alterations (*SI Appendix*, *SI Text*); and (4) the sediment cores used in this study have good age models with no stratigraphic disturbance. To further validate the Fe-bound P proxy at the SEPR sites, we analyzed the Fe and P content in OC-73–3-20 spanning the past ∼40 ky. Independent reconstructions of deep-ocean phosphate concentrations from foraminifera Cd/Ca data suggest little change on this timescale (18, 19). Indeed, the Fe and P content display a strong correlation in OC-73–3-20 with only one slope, consistent with the Cd/Ca reconstructions and supporting the use of the Fe-bound P proxy on longer timescales.

In DSDP 598, the Fe and P contents display tight correlations during each epoch/stage (*SI Appendix*, Fig. S3, *r*^2^ > 0.97). However, the slopes of this Fe-P correlation decreased dramatically over the past 10 My—from ∼94 in the Tortonian to ∼31 in the Messinian and ∼19 in the Pleistocene (*SI Appendix*, Fig. S3). The Pleistocene samples in DSDP 598 display a Fe-P slope only a little higher than that of nearby core OC-73–3-20 (19.4 ± 1.76 versus 14.87 ± 1.52), supporting the robustness of this proxy for deep-water phosphate concentrations. To quantify the deep-ocean [PO_4_^3−^] for the past 12 My, we computed the running slopes of the Fe-P records for a window size of 1.5 My and converted them to the deep-ocean [PO_4_^3−^] by calibrating our late Pleistocene observations with the modern [PO_4_^3−^] value at the study site (see *SI Appendix*, Fig. S6 and *SI Text*). Over the entire interval, the correlation coefficients of Fe-P slopes remain higher than 0.85 (*SI Appendix*, Fig. S6), consistent with the proximity of this site to the ridge and signifying that hydrothermal processes have persistently been the dominant control on the Fe and P content of the sediments captured by this core. We applied a depth correction to the reconstructed [PO_4_^3−^], because the DSDP 598 site has been migrating to deeper water as it moves further off axis. However, this correction causes negligible changes to the results (i.e., <0.05 μM) because [PO_4_^3−^] is largely homogeneous below 3,000 m in the Pacific Ocean (Fig. 1).

**Fig. 2.**
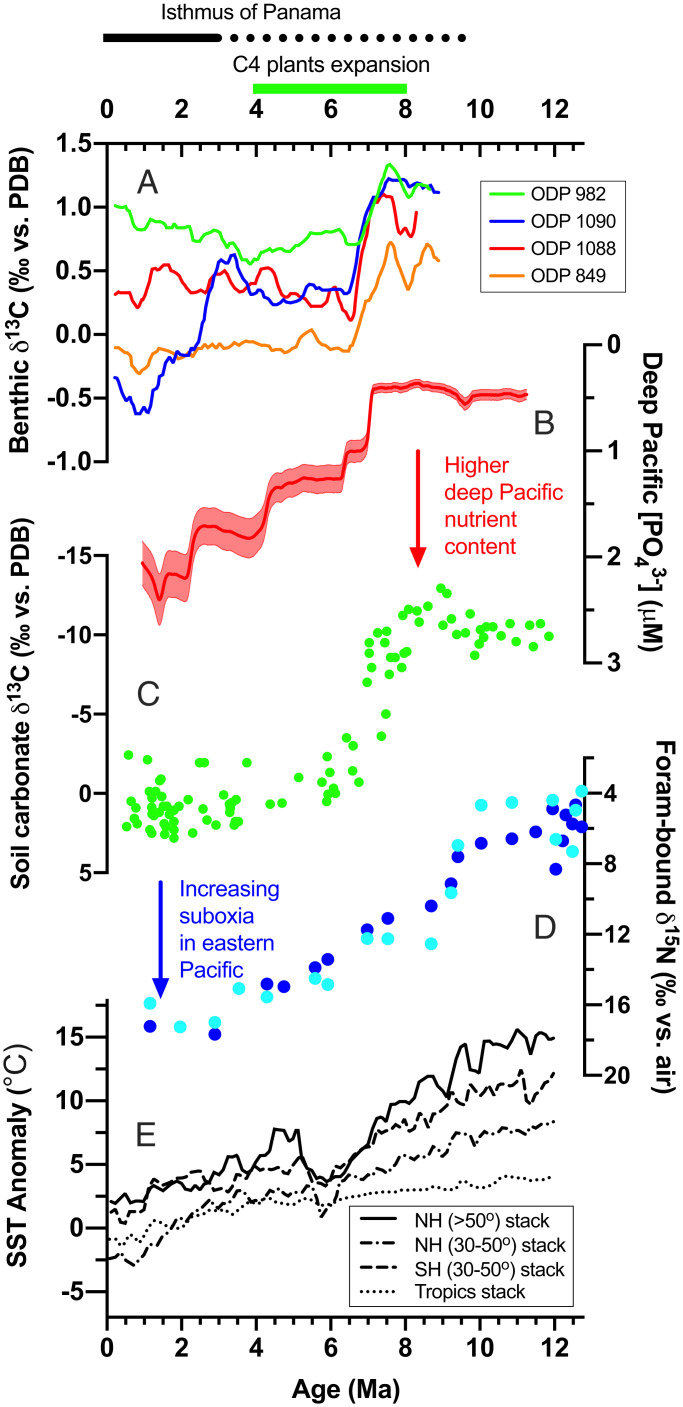
Reconstructed deep-ocean [PO_4_^3−^] and ODZs δ^15^N records over the past 12 My. (*A*) Benthic δ^13^C compilation (30); (*B*) reconstructed [PO_4_^3−^] based on the P and Fe content in DSDP 598 (this study); (*C*) soil carbonate δ^13^C from northern Pakistan (40); (*D*) foraminifera-bound δ^15^N from DSDP 598 (this study; blue: 125 to 250 μm fraction; cyan: >250 μm fraction); and (*E*) Ses Surface Temperatur (SST) compilation over the past 12 My (37). The timing of C4 plant expansion and closure of the Isthmus of Panama are indicated at the *Top*, with the dashed line indicating uncertainty (27). The error envelope (1σ) of the [PO_4_^3−^] record was generated using Monte Carlo simulation, accounting for all errors (*SI Appendix*, Fig. S6 and *SI Text*).

In summary, we generated records of foraminifera-bound δ^15^N and deep-ocean [PO_4_^3−^] in the South Pacific since 12 My (Fig. 2). The most prominent feature of the foraminifera-bound δ^15^N record is a gradual and large increase of 10 to 11‰ since the late Miocene, from average δ^15^N values of ∼6‰ before 9 My to 16 to 17‰ by 1 My. With an offset of 2 to 3‰ between Holocene foraminifera-bound δ^15^N and subsurface nitrate δ^15^N (*SI Appendix*, Fig. S2 and *SI Text*), the subsurface nitrate δ^15^N in the eastern Pacific before 9 My would have been 3 to 4‰, indicating much smaller ODZs at that time. Thus, the late Miocene marks a dramatic intensification of South Pacific ODZs. In parallel, the deep-ocean [PO_4_^3−^] record displays a large increase since the late Miocene. Before 8 My, the [PO_4_^3−^] in the deep Pacific was only ∼0.5 μM—a value that is less than 50% of the minimum [PO_4_^3−^] in the modern open ocean. From 8 to 6 My, the deep-Pacific [PO_4_^3−^] increased by 0.8 μM, representing the largest increase over the past 12 My. This increase is corroborated by a benthic foraminifera Cd/Ca record from the west Pacific (*SI Appendix*, Fig. S7) and coincides with similar changes in global benthic foraminifera δ^13^C records, as well as the expansion of C4 plants on land exemplified by the soil carbonate δ^13^C record from northern Pakistan (Fig. 2). The δ^15^N change appears to have preceded the increase in [PO_4_^3−^] change by ∼1–2 My (Fig. 2). Because the δ^15^N and [PO_4_^3−^] records were generated on the same samples, this 1–2 My lag is not due to age model uncertainties. Rather, the timing of the δ^15^N change is more consistent with the sea-surface temperature (SST) records (Fig. 2), which showed that cooling may have preceded the δ^13^C and [PO_4_^3−^] changes. It is thus likely that the early change in the δ^15^N record before 8 My was related to global cooling, such as through high-latitude ocean stratification and its impact on deep-ocean O_2_ concentrations (20). However, the effect of global cooling on the expansion of ODZs since the late Miocene is only secondary, as indicated by the model results below. Nevertheless, most of the δ^15^N increase occurred in concert with the [PO_4_^3−^] increase.

Although our study site is well within the oligotrophic waters of the South Pacific today (Fig. 1), one may wonder whether the equatorial Pacific high-nutrient tongue extended further south in the past, causing incomplete surface nitrate consumption and lowering foraminifera-bound δ^15^N at our study sites in the early part of the record. However, the effect of the high-nutrient tongue would be to increase, not decrease, foraminifera-bound δ^15^N at the latitudes of our core sites. This is the case because the residual nitrate and particulate nitrogen at the margins of the high-nutrient tongue are elevated in δ^15^N due to prior nitrate consumption (with isotopic fractionation) closer to the equator, coupled with flow away from the equator (13, 21, 22). Thus, the observed low foraminifera-bound δ^15^N values before 8 My require a smaller ODZs and an equatorial nutrient tongue that was either similar to or smaller than that of today. Indeed, the lower whole ocean [PO_4_^3−^] that we infer for the late Miocene may well lead to a contraction in the equatorial Pacific nutrient tongue. Regardless of past changes in the equatorial nutrient tongue, the foraminifera-bound δ^15^N values of ≤8‰ before 9 My could not have been reached without a smaller ODZs at that time.

Our deep-ocean [PO_4_^3−^] record was derived from the South Pacific, and while this reflects the largest body of water on the planet, it is important to consider whether this record may be caused by a redistribution of phosphate between the Atlantic and Pacific without requiring a change in the global ocean’s nutrient content. It has long been thought that the formation of the Isthmus of Panama would have changed ocean circulation (23–25) and affected the distribution of nutrients between the Atlantic and Pacific Oceans (26). Before the closure of the Central American Seaway (CAS), a significant amount of North Atlantic deep water could flow through the CAS to the Pacific, which has been shown in models to decrease the nutrient content of the deep Pacific (25, 26). Although the timing of the formation of the Isthmus of Panama is debated, most paleoceanographic data point to a timing more recent than 5 My (27–29). Furthermore, the reconstructed [PO_4_^3−^] before 8 My was only 0.5 μM, less than 50% of minimum [PO_4_^3−^] in the modern deep ocean. A redistribution of nutrients between the Atlantic and Pacific Oceans would not be adequate to cause such low [PO_4_^3−^]. Consequently, the increase in deep Pacific [PO_4_^3−^] during the late Miocene is best explained as an increase in the mean [PO_4_^3−^] of the global ocean. This interpretation is supported by the δ^13^C gradient reconstructed between the Atlantic and Pacific Oceans, which showed minimal change during this time (30).

**Fig. 3.**
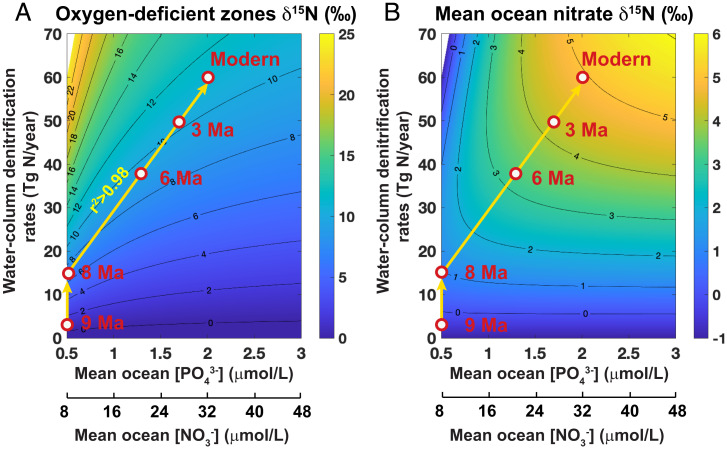
Box model estimation of the response of water-column denitrification rate to mean ocean [PO_4_^3−^] since 9 My. Model output of the δ^15^N of ODZs nitrate (*A*) and of mean ocean nitrate (*B*) as a function of water-column denitrification rate and mean ocean nutrient content (with assumed Redfield ratio of [NO_3_^−^]/[PO_4_^3−^] = 16). The contours were generated using a two-box model (*SI Appendix*, Figs. S8–S10 and *SI Text*). The symbols and arrows indicated the reconstructed ODZs δ^15^N and deep-ocean [PO_4_^3−^] from DSDP 598 (see Fig. 2). The calculated water-column denitrification rate displays a strong linear correlation (*r*^2^ > 0.98) with the mean ocean [PO_4_^3−^], with a slope of ~31 Tg N/y/μM [PO_4_^3−^]. The foraminifera-bound δ^15^N record reveals a ∼11‰ change since 9 My, with a 5 to 6‰ increase from 9 to 8 My and another 5‰ from 8 My to the Pleistocene. The [PO_4_^3−^] concentration did not change from 9 to 8 My, but displays a three- to four-fold rise from 8 My to the Pleistocene.

We used an idealized two-box model to quantify the extent of suboxia and its relationship with the rising nutrient content of the ocean since the late Miocene (*SI Appendix*, Fig. S8 and *SI Text*). In this model, the combined ODZs (<1% of total ocean volume) are separated from the rest of the ocean (mean ocean). Fixed nitrogen (i.e., “biologically available nitrogen”) is lost from the ODZs through water-column denitrification and from the mean ocean through sedimentary denitrification. Fixed nitrogen is added to the ocean through N_2_ fixation at a rate equivalent to that of total denitrification. Assuming a constant sedimentary denitrification rate of 130 Tg N/y (31) and a constant Redfield ratio (mean ocean [NO_3_^−^]/[PO_4_^3−^] = 16), we derive the relationships between ODZs nitrate δ^15^N, water column denitrification rate, and mean ocean nutrient content (contours in Fig. 3). The reconstructed [PO_4_^3−^] and δ^15^N records are used to calculate the rates of water-column denitrification (suboxia) from the late Miocene to the present (symbols and arrows in Fig. 3).

From 9 to 8 My, the mean ocean nutrient content did not change, while ODZs δ^15^N increased by 5 to 6‰. However, the model indicates that this 5 to 6‰ increase reflected only a modest increase in water-column denitrification rate (∼10 Tg N/y, or ∼17% of modern value). This is mainly because of the low nutrient content and near absence of water-column denitrification before 9 My. In such an ocean, the ODZs δ^15^N is highly sensitive to small changes in water-column denitrification rates (Fig. 3). Since 8 My, both water column denitrification and mean ocean nutrient content gradually increased to their modern values, with the largest change between 8 and 6 My. The evolution of the water-column denitrification rate and mean ocean nutrient content were linearly well correlated (*r*^2^ > 0.98) since 8 My (Fig. 3), with a slope of ∼31 Tg N/y/μM [PO_4_^3−^]. This sensitivity of water-column denitrification rates to mean ocean nutrient change falls within the range of estimates from more complex biogeochemical models (7, 32). The strong linear correlation between water-column denitrification rate and mean ocean nutrient content since 8 My suggests that most of the ODZs expansion was driven by the nutrient increase. Indeed, the nutrient content of the ocean plays a dominant role in the biological pump, the strength of which is inversely related to the O_2_ concentration of the ocean interior through export production and microbial respiration (33, 34). Consistent with this expectation, an increase in productivity metrics have been observed since the late Miocene, with a “biogenic bloom” between 8 and 6 My (35–37). Although the decreasing SST and increasing O_2_ production may have supplied more O_2_ to the ocean since the late Miocene (*SI Appendix*, *SI Text*), the rising oceanic nutrient content and resulting higher productivity appear to have overwhelmed these effects, driving ocean deoxygenation over the past 8 My. Whereas studies of global warming have tended to cast ocean O_2_ content as dependent on climate and ocean circulation (1, 2), our study indicates that the modern ODZs are underpinned by a historically high oceanic concentration of phosphate.

The rise in the water-column denitrification rate since the late Miocene has important implications for the rate of N_2_ fixation in the global ocean. As the residence time of fixed nitrogen is only 3 to 5 ky in the ocean (38), the rate of N_2_ fixation is set equal to the rate of total denitrification in the model such that fixed N losses and inputs are always balanced on million-year timescales. As a result, the N_2_ fixation rate has increased since the late Miocene in the model, in response to the increasing watercolumn denitrification rate. In today’s ocean, the highest N_2_ fixation rates are observed in the western Pacific, with little N_2_ fixation near the Pacific ODZs (39). Our reconstructed ODZs history would imply that the N_2_ fixation rate in the western Pacific before 8 My was lower than today.

According to our data, the late Miocene experienced the largest jump in ocean [PO_4_^3−^]. The late Miocene interval marks a time of substantial change in both the climate and biosphere. On land, plants using the C4 photosynthesis pathway—a metabolic strategy for concentrating CO_2_ in plant tissues—expanded, changing landscapes and ecosystems in subtropical and tropical regions (40). Africa and East Asia became more arid, and large deserts such as the Sahara emerged (41). These vegetation and landscape shifts also coincided with the divergence between lineages that would give rise to humans and chimpanzees (42). In the oceans, SST records from both hemispheres show a dramatic decline from 7 to 4 My, with a concomitant increase in the meridional temperature gradient (37). These environmental changes have long been linked to evidence for an increase in ocean productivity (36) and a strengthening of the biological carbon pump of the oceans (30).

One striking feature of the [PO_4_^3−^] record is that the late Miocene jump coincided with the global benthic δ^13^C decline and the expansion of C4 plants on land, implying a common driver of these changes (Fig. 2). Although recent studies suggested that the timing of the expansion of C4 plants may have been different at different places, most of these changes occurred between 8 and 6 My (43). One hypothesis proposed for the late Miocene benthic δ^13^C decline is that the expansion of C4 plants changed the land surfaces to less dense grasslands, triggering greater soil erosion and higher soil organic matter input to the oceans (36). The subsequent remineralization of low-δ^13^C soil organic matter in the ocean would have caused a decline in the δ^13^C of the mean ocean carbon reservoir (37). Because soil organic matter contains a significant amount of P, the remineralization of soil organic carbon would have also increased the P input into the ocean, causing a transient imbalance between the source and sinks of oceanic P reservoir. The net result would be a rise in the mean ocean’s nutrient content. Our reconstructed deep-ocean [PO_4_^3−^] rose by ∼0.8 μM from 8 to 6 My, consistent with the soil erosion hypothesis.

The deep-ocean [PO_4_^3−^] continued to rise after 6 My, however, while the global benthic and soil δ^13^C records stabilized at 6 My (Fig. 2). Also, the soil organic matter reservoir is a temporary carbon reservoir on geological timescales, so faster soil erosion alone cannot sustain the accelerated delivery of nutrients to the ocean. It is thus unlikely that the nutrient increase after 6 My was caused by the same soil erosion mechanism due to C4 expansion. We propose that the increase in the deep-Pacific [PO_4_^3−^] after 6 My was caused by an increased weathering rate. Recent work suggests that the global weatherability increased since the mid- to late Miocene due to the emergence of the Southeast Asian islands as a result of arc-continent collision (44). If correct, then such an increase in weathering could have contributed to the observed increase in the deep ocean [PO_4_^3−^], particularly the gradual deep-ocean [PO_4_^3−^] increase after 6 My.

Beyond the expansion of marine ODZs, the increase in the nutrient content of the oceans has important implications for the atmospheric *p*CO_2_. The extra nutrients would have increased primary productivity in much of the world’s oceans, sequestering additional CO_2_ from the atmosphere in the deep ocean. This ocean nutrient content mechanism was originally proposed to explain the *p*CO_2_ variation seen on glacial-interglacial timescales (33). Although no convincing evidence has emerged for such a change in glacial-interglacial timescales, our results suggest that this mechanism was at work earlier in the history of the Earth. The ∼0.8-μM [PO_4_^3−^] increase in the late Miocene could have caused an abrupt ∼60 ppm decrease in atmospheric *p*CO_2_, depending on the responses of pelagic CaCO_3_ production and high latitude productivity (45). Since 6 My, increased weathering of P and of alkalinity (46) may have worked together to further reduce atmospheric *p*CO_2_. Although Miocene-age *p*CO_2_ reconstructions have yielded mixed results (*SI Appendix*, Fig. S7), some studies have shown a *p*CO_2_ decline during this interval (47, 48). If confirmed, such a decrease in atmospheric CO_2_ would help to explain the observed global cooling and other environmental and evolutionary changes since the late Miocene, such as the increase in aridity in Asia and Africa and the evolution and expansion of C4 photosynthesis.

## Materials and Methods

### Materials.

OC-73–3-20 (19.25 °S, 113.58 °W) was drilled in 1973 by *R/V Oceanographer* at a water depth of 3,081 m from ∼8 km west of the SEPR ridge axis. The samples were requested from the Oregon State University Core Repository. The age model of this core since 40 ky was described in a previous study (12). Due to its short length and time span captured, this core was used to ground-truth the modern and recent P-Fe correlations and foraminifera-bound δ^15^N.

DSDP 598 (19.00 ^o^S, 124.68 ^o^W) was drilled in 1983 by *R/V Glomar Challenger* at a water depth of 3,699 m from the SEPR. The samples were obtained from the Gulf Coast Repository through the International Ocean Discovery Program. An age model was produced in the DSDP Initial Report, with updates to a new timescale (*SI Appendix*, Fig. S4) (49). In the modern Ocean, this site is ∼1,150 km to the west of OC-73–3-20. The off-axis spreading rate in SEPR was estimated to be 77 mm/y (12), which worked out to a distance of ∼770 km over 10 My. Thus, in the late Miocene, DSDP 598 would have been adjacent to today’s location of OC-73–3-20, and consequently, OC-73–3-20 provided a useful modern analog site for longer time series developed from DSDP 598.

### Methods.

#### Foraminifera-bound δ^15^N.

Foraminifera-bound δ^15^N measurements were performed on both DSDP 598 (0-12 My) and OC-73–3-20 (0 to 40 ky) samples at Boston College and Princeton University following the protocols previously described in refs. 14, 50, and 51, with a few modifications. The bulk sediment was sieved into three size fractions: >250, 125 to 250, and <125 μm. The >250 μm and 125 to 250 μm fractions were transferred into 15-mL centrifuge tubes and chemically cleaned twice to remove clays and Fe/manganese (Mn) oxides before examination of the foraminifera under a microscope. Briefly, 10 mL of 2% sodium polyphosphate solution (pH ∼8) was added into each sample and sonicated for 5 min. After sonication, the samples were rinsed three times with MilliQ water and 10 mL of dithionite and citric acid solution (pH ∼8) was added into each sample. The samples were then placed in an 80 °C water bath for 1 h to allow for the reduction and removal of Fe/Mn oxides, which are abundant in the SEPR samples because of the hydrothermal input. These sediment cleaning steps were repeated once. Then, the samples were rinsed three times using MilliQ water and dried in an oven at 60 °C.

After drying, the sediment samples were examined via light microscopy and were entirely composed of planktonic foraminifera. An aliquot of the foraminifera samples was weighed out, gently crushed, and placed in a 15-mL centrifuge tube. The sodium polyphosphate and dithionite clean steps were performed again on the crushed foraminifera to further remove any potential residual clay and Fe/Mn oxides coated on the surface of the foraminifera. After the reductive cleaning, 10 mL sodium hypochlorite (10 to 15% available chlorine) was added to each sample, and the centrifuge tubes were placed on a shaker for 24 h. This step was done to remove any exposed reducing nitrogen on the surface of the foraminifera. After the oxidative cleaning, the samples were rinsed 3 times with MilliQ water, transferred into a precombusted 12-mL glass vial, and oven dried at 60 °C. In each batch of analyses, three replicates of a coral standard (CBS-I) with known δ^15^N value were included following the same cleaning procedures as described for the samples, serving as quality control of the cleaning protocol.

After drying, 3 to 10 mg of each foraminifera sample was weighed into a precombusted 4-mL glass vial and digested with 50 μL of 4 N HCl. Then, 1 mL of fresh-made persulfate reagent (1 g recrystallized low-N potassium persulfate and 2 g American Chemical Society [ACS] grade NaOH dissolved in 100 mL of MilliQ water) was added into each vial. Procedural/reagent blanks as well as glycine standards with known δ^15^N values (US Geological Survey [USGS]40 and USGS65) were included at this step, serving as quality control of the oxidation protocol. The vials were capped and autoclaved for 1 h to ensure the complete oxidation of the foraminifera-bound organic nitrogen. After oxidation, the samples were centrifuged for 10 min at 6,000 rpm. Then, the supernatant was transferred into a new precombusted 4 mL glass vial, with the pH of the supernatant adjusted to neutral before analyses of the resulting nitrate.

The nitrate concentration was analyzed using the chemiluminescence method (52), followed by the isotopic analyses of the nitrate using the denitrifier method (53, 54). Coral standards, procedural/reagent blanks, and glycine standards (USGS40 and USGS65) were analyzed along with the samples, allowing for the correction of the blanks. The N blanks were typically ∼0.2 μmol/L in an oxidized sample, representing 1 to 5% of total N in the samples. The analytical precision of the protocol—determined by the coral standard in each batch of analyses (CBS-I)—was ∼0.2‰ (1σ).

#### P and Fe content.

The P and Fe contents in OC-73–3-20 were analyzed using an INAM Expert 3L desktop X-ray fluorescence at the California Institute of Technology. Two USGS standards (MAG-1 and SBC-1) were used to calibrate the P content of the samples. The average analytical errors were 4% (1σ) for P content and 0.3% (1σ) for Fe content. The P and Fe content data in DSDP 598 were obtained from the DSDP initial reports (55).

#### Validation of the Fe-P proxy in OC-73–3-20.

To further validate the hydrothermal Fe-P proxy in the SEPR, we analyzed the Fe and P content in OC-73–3-20 spanning the past ∼40 ky (*SI Appendix*, Fig. S3). This core is located close to the ridge axis and characterized by extremely high Fe and P contents due to the hydrothermal processes that sourced much of the sediment. Although both the Fe and P content displayed fluctuations down core, they exhibited a strong correlation (*r*^2^ = 0.95, least-squares regression), with a slope of 14.87 ± 1.52 (*SI Appendix*, Fig. S3). This tight relationship indicated that (1) the P content in the SEPR hydrothermal sediments is controlled by the deposition of Fe oxyhydroxides and (2) the phosphate concentration at this site has remained largely unchanged since the last glacial period—a feature that also matched previous reconstructions of deep-Pacific [PO_4_^3−^] based on measurements of the Cd/Ca in benthic foraminifera (18).

#### Conversion of P and Fe content to phosphate concentration (0 to 12 My).

The Fe:P (or P:Fe) ratio has been used previously to indicate potential past phosphate concentration changes in seawater (see, for example, ref. 49). However, Fe:P alone is not an ideal indicator for phosphate concentrations in the past because both the background Fe and P changes caused by idiosyncratic changes in the nonhydrothermal detrital components (i.e., the intercept of Fe-P crossplots) could lead to a change in Fe:P. Rather, it is the slope of the Fe-P plot that is more directly linked to the phosphate concentration changes in hydrothermal sediments (17). For a given phosphate concentration in the deep ocean, both the Fe and P content may change with the hydrothermal activities, but the slope of the Fe-P plots would remain unchanged (17). We found that the Fe-P slope in OC-73–3-20 from 0 to 40 ky was only a little higher than the Fe-P slope of Pleistocene samples in DSDP 598 (*SI Appendix*, Fig. S3, 19.4 ± 1.76 versus 14.87 ± 1.52), validating the robustness of this correlation.

Crossplots of Fe versus P content in DSDP 598 (*SI Appendix*, Fig. S3) for 4 different time intervals (Pleistocene, Pliocene, Messinian, and Tortonian) all showed tight correlations (*r*^2^ > 0.97). However, the slopes of these crossplots were remarkably different, indicating that phosphate concentrations have been changing with time at the study site. To quantitatively convert the Fe-P slopes to the phosphate concentration in the past, we calculated the running slopes and correlation coefficients with a few choices of regression time windows (*SI Appendix*, Fig. S6). Then, we converted the slopes to the phosphate concentration by assigning the modern [PO_4_^3−^] value (2.5 μM) at the site of OC-73–3-20 (Fig. 1). We found that with a window size of 1.5 My, the correlation coefficients throughout the entire time interval were higher than 0.85 (*SI Appendix*, Fig. S6). Thus, we chose this window size for the calculation of phosphate concentration since 12 My (Fig. 2). The error envelopes were then calculated using a Monte Carlo approach that accounted for all of the errors in the regression.

## Supplementary Material

Supplementary File

## Data Availability

All study data are included in the article and/or supporting information.
