# Supplementary material for: Oceanic nutrient rise and the late Miocene inception of Pacific oxygen-deficient zones
Source: Proc Natl Acad Sci U S A. 2022 Nov 2;119(45):e2204986119. doi: 10.1073/pnas.2204986119 (PMC9659387; doi:10.1073/pnas.2204986119)
Supplement: Supplementary File [file pnas.2204986119.sapp.pdf]

Supporting Information for

Oceanic nutrient rise and the late Miocene inception of Pacific oxygen deficient zones

Xingchen Tony Wang, Yuwei Wang, Alexandra Auderset, Daniel M. Sigman, Haojia Ren, Alfredo Martínez-García, Gerald H. Haug, Zhan Su, Yi Ge Zhang, Birger Rasmussen, Alex L. Sessions, Woodward W. Fischer

Xingchen Tony Wang
Email: xingchen.wang@bc.edu

This PDF file includes:

Supporting text
Figures S1 to S12
SI References

Other supporting materials for this manuscript include the following:

Datasets S1 to S2

Supporting Information Text

DSDP 598 as an ideal site for reconstructing the history of South Pacific ODZs

The modern Pacific ODZs are mainly located in the eastern tropical Pacific (Fig. S1A). In these ODZs, water-column denitrification preferentially consumes ^{14}N relative to ^{15}N, leaving behind high-$\delta^{15}N$ nitrate (1). These remaining nitrate exchanges with the rest of the ocean, raising the whole ocean $\delta^{15}N$ (2). Regions closer to the ODZs or more directly exchanging with the ODZs are characterized by high $\delta^{15}N$ in the thermocline water (Fig. S1B). As a result, thermocline nitrate $\delta^{15}N$ is a robust indicator for the extent of ODZs. Previous studies have demonstrated that, in regions where nitrate consumption is complete at the surface (e.g., oligotrophic gyres), foraminifera-bound $\delta^{15}N$ reliably record the thermocline nitrate $\delta^{15}N$ (3). In ocean regions with incomplete nitrate consumption at the surface (e.g., upwelling zones), foraminifera-bound $\delta^{15}N$ is also affected by the degree of nitrate consumption as phytoplankton preferentially consumes ^{14}N relative to ^{15}N as well (4).

Thus, when selecting a site to reconstruct the history of ODZs using foraminifera-bound $\delta^{15}N$, two requirements must be satisfied: (1) the thermocline nitrate at the site should be dominantly sourced from the ODZs; and (2) nitrate consumption should be complete at the surface such that the foraminifera-bound $\delta^{15}N$ record is not complicated by potential changes in the degree of nitrate consumption. Although there are many available sediment cores in the eastern tropical Pacific, most of the sites are influenced by both the changing extent of ODZs and varying degree of surface nitrate consumption, which has complicated the interpretation of previous $\delta^{15}N$ records on glacial-interglacial timescales (5). To avoid this problem, we have chosen the SEPR sites (DSDP 598 and OC 73-3-20) in this study because they are located within the oligotrophic zone of the south Pacific (Fig. 1). In addition, previous nearby nitrate $\delta^{15}N$ measurements

indicated that the thermocline nitrate at these sites is dominantly sourced from the ODZs (Fig. S1C). Therefore, DSDP 598 is ideal for reconstructing the long history of ODZs in the south Pacific.

Potential impacts of diagenesis on the DSDP 598 records

The role of diagenesis must be considered when interpreting sedimentary records of any age. The high concentration of iron/manganese oxides in DSDP 598 might make it vulnerable to the input of organic carbon, which would reduce the iron/manganese oxides and alter the Fe-P relationship (Fig. S3). However, the location of DSDP 598 has always been within the oligotrophic gyre of the South Pacific Ocean. Due to the low productivity in the surface ocean, the organic carbon content in DSDP 598 (<0.1wt %) is two orders of magnitude lower than the Fe content (Fig. S4 and S11), such that any iron/manganese reduction by organic matter would cause negligible impact on the Fe content. As these iron-rich sediments are not saturated with phosphate(6), the diagenetic loss of phosphate to seawater should also be minimal. Indeed, a previous study of the Fe and P speciation changes in this core also suggested minimal loss of Fe and P from the sediments since its deposition, and the Fe-P relationship changes are best explained by deep-ocean phosphate concentration changes (7).

Similarly, diagenesis might also impact foraminifera-bound $\delta^{15}N$ if the foraminifera shells were partially dissolved and a portion of the foraminifera-bound nitrogen was lost. The low organic carbon content in DSDP 598 also minimized the carbonate dissolution that could have occurred. Indeed, examination of these foraminifera under microscope did not display any signs of dissolution (e.g., etching of chamber walls). In addition, any nitrogen loss and the associated isotopic fractionation would typically increase the $\delta^{15}N$ of the remaining nitrogen such that a negative correlation is expected between foraminifera-bound N content and $\delta^{15}N$ (8). However,

our data in DSDP 598 did not show such a negative correlation (Fig. S12), indicating that diagenesis did not impact the measured $\delta^{15}N$ values.

Potential influence of foraminifera species changes on the foraminifera-bound $\delta^{15}N$ record

In the $\delta^{15}N$ records, we analyzed the mixed foraminifera species in two size fractions (i.e., 125 to 250 μm and >250 μm fraction). One might wonder if changes in foraminifera species over time might be responsible for the long-term $\delta^{15}N$ trend. However, the $\delta^{15}N$ differences among difference foraminifera species at a given site are typically <3‰ (3, 9), much less than the observed >10‰ change since late Miocene time. In addition, it has been shown that the size-specific foraminifera-bound $\delta^{15}N$ records agreed well with the species-specific foraminifera-bound $\delta^{15}N$ records at the same site (10). In our records, the 125 to 250 μm and >250 μm records also agree well with each other, further indicating that the long-term $\delta^{15}N$ trend cannot be explained by the changes in foraminifera species.

Other potential drivers of ocean oxygen content since late Miocene time

One might wonder if an increase in the ocean's nutrient content could cause an increase in organic carbon burial rates, which would lead to higher O_2 concentrations in the atmosphere and the oceans, and thus work to curb the expansion of ODZs. However, the size of the O_2 reservoir in the atmosphere is so large that it is unlikely to have increased by more than 10% during the Neogene (11). Global cooling could have worked to increase the supply of O_2 to the ocean through greater solubility in seawater. However, a decrease of global average SST of 6 ^oC (Fig. 2) from 25 ^oC to 19 ^oC (12) since 10 Ma would only cause a ~10% increase in dissolved O_2 concentration in the surface ocean (13). With these two factors combined, Henry's Law would suggest that the supply of O_2 to a more productive, colder ocean to increase by no more than

~20% since late Miocene time. In addition, the supply of O_2 to the eastern Pacific ODZs by changing ocean circulation is not necessarily enhanced by global cooling (5, 14). Overall, the higher O_2 supply could be easily overwhelmed by the higher oxygen consumption due to higher oceanic nutrient content and resulting rise in productivity, including in the upwelling zones that overlap with the ODZs (Fig. S7).

A two-box model for the evolution of oceanic nutrient content and N isotopic budget since 12 Ma

We built a simple two-box mathematical model to calculate the global suboxia change since 12 Ma, using constraints from our observed foraminifera-bound $\delta^{15}N$ and phosphate concentration records. The topology of the model is shown in Fig. S8. In this model, the ODZs box (<1% of total ocean volume) is separated from the rest of the ocean and the communication of the ODZs box with the rest of the ocean is described by a mixing term (υ). The input of fixed N to the entire ocean is through N_2 fixation (ϕ_1), which has a fixed $\delta^{15}N$ of -1‰ and no isotopic fractionation. There are two nitrogen loss pathways in the model: water-column denitrification and benthic denitrification. Water-column denitrification is confined to the ODZs box, with an isotope fractionation of 25‰ (ϕ_3). Benthic denitrification happens in the mean ocean box with no isotope fractionation (ϕ_2).

At steady state, we derived the following equations:

$$\phi_1 = \phi_2 + \phi_3 \quad (1)$$

$$\upsilon\,(N_0 - N_d) = \phi_3 \quad (2)$$

$$(\delta_f - \varepsilon_1)\phi_1 = (\delta_0 - \varepsilon_2)\phi_2 + (\delta_d - \varepsilon_3)\phi_3 \quad (3)$$

$$\delta_0 \upsilon N_0 - \delta_d \upsilon N_d = (\delta_d - \varepsilon_3)\phi_3 \quad (4)$$

Solving for δ_0 and δ_d, we found:

$$\delta_0 = \delta_f + \varepsilon_3 \frac{\phi_3}{\phi_1} - \varepsilon_3 \frac{\phi_3}{\phi_1} \frac{\phi_3}{\upsilon N_0} \quad (5)$$

$$\delta_d = \delta_f + \varepsilon_3 \frac{\phi_3}{\phi_1} + \varepsilon_3 \left(1 - \frac{\phi_3}{\phi_1}\right) \frac{\phi_3}{\upsilon N_0} \quad (6)$$

Let:

$$f = \frac{\phi_3}{\phi_1} \quad (7)$$

$$g = \frac{\phi_3}{\upsilon N_0} = 1 - \frac{N_d}{N_0} \quad (8)$$

And δ_0 and δ_d could be rewritten as:

$$\delta_0 = \delta_f + \varepsilon_3 f - \varepsilon_3 f g \ (9)$$

$$\delta_d = \delta_f + \varepsilon_3 f + \varepsilon_3 (1 - f) g = \delta_0 + \varepsilon_3 g \ (10)$$

These equations were then used to generate the contour plots in Fig. 3 and Fig. S9, by assigning modern values for certain parameters and making the following assumptions: (1) The mean

ocean nitrate and phosphate concentrations follows Redfield ratio such that $N_0 = 16P_0$, as both N and P have residence times of less than 100,000 years in the oceans, this assumption is unlikely to have been violated since 12 Ma; (2) The global benthic/sedimentary denitrification rate remained unchanged since 12 Ma, with a modern value of 130 Tg N/year (15); (3) Water-column denitrification rate in the modern ocean was set to 60 Tg N/year (15), and was allowed to change in the model; (4) A value of 17 Sv was chosen for the mixing rate (υ) by fitting the modern values of mean ocean nitrate concentration and $\delta^{15}N$ ($N_0 = 32\ \mu mol/L; \delta_0 = 5‰$); this mixing rate was held constant. This value is similar to the mixing rate in the 8-box model presented in (16), which was used to calculate the marine N budget on glacial-interglacial timescales.

With these assumptions and our ODZs $\delta^{15}N$ and mean ocean phosphate concentration constraints (Fig. 2), we calculated the water-column denitrification rates as well as the mean ocean nitrate $\delta^{15}N$ change since 12 Ma (Fig. 3).

In addition, we have tested the sensitivity of the model to changing benthic denitrification rates (Fig. S10). The delivery rate of organic matter to the ocean seafloor is an important factor affecting benthic denitrification rates (17). In a low-nutrient ocean, it is expected that benthic denitrification rates would have also been lower. Thus, we also made the benthic denitrification rates linearly scale with the mean ocean phosphate concentration (equation (11)), which dominantly controls the ocean's export production. Despite this new parameterization, a strong correlation was still observed between the water-column denitrification rate and mean ocean phosphate concentration (Fig. S10), with a slightly higher slope than Fig. 3A (35 vs. 31 Tg N/year/μM [PO_4^{3-}]).

$$\phi_2 = \frac{P_0}{P_0(modern)} \times 130\ Tg\ N/year \quad (11)$$

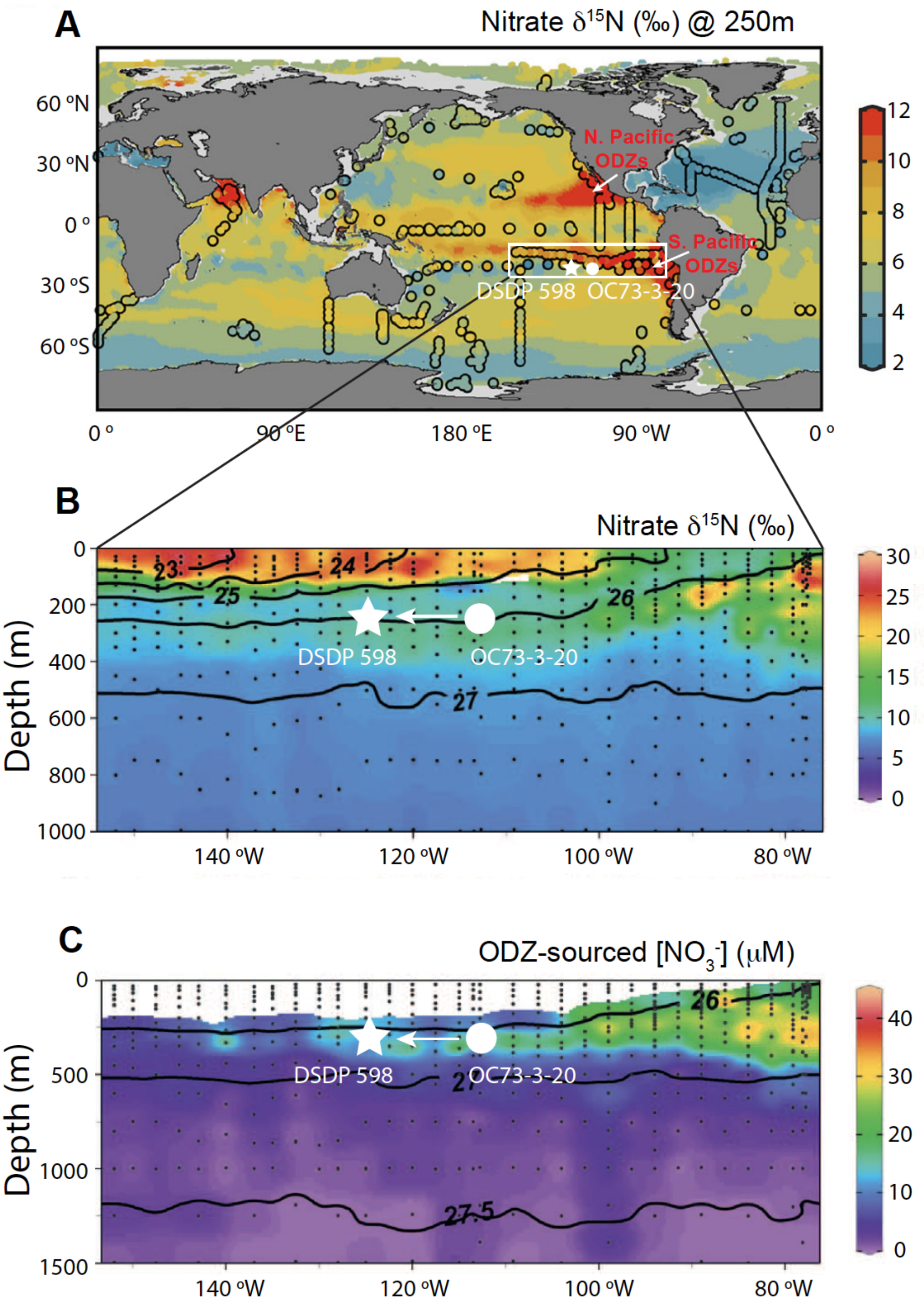
A
Nitrate δ15N (‰) @ 250m
N. Pacific ODZs
S. Pacific ODZs
DSDP 598
OC73-3-20
60 °N
30 °N
0 °
30 °S
60 °S
0 °
90 °E
180 °E
90 °W
0 °
12
10
8
6
4
2
B
Nitrate δ15N (‰)
Depth (m)
0
200
400
600
800
1000
23
24
25
26
27
DSDP 598
OC73-3-20
140 °W
120 °W
100 °W
80 °W
30
25
20
15
10
5
0
C
ODZ-sourced [NO3-] (μM)
Depth (m)
0
500
1000
1500
26
27
27.5
DSDP 598
OC73-3-20
140 °W
120 °W
100 °W
80 °W
40
30
20
10
0

Fig. S1 (A) A map showing modern thermocline nitrate $\delta^{15}N$ at 250 m and our study sites (Figure adapted from (18)). Red areas with very high $\delta^{15}N$ indicate ODZs with active water-column denitrification. The high-$\delta^{15}N$ signal in the east Pacific ODZs propagates westward through ocean circulation, increasing the thermocline nitrate $\delta^{15}N$ in the subtropical gyres (e.g., at our study sites). The circles indicate actual nitrate $\delta^{15}N$ measurements while the map was generated from a neural network-based climatology. (**B**) Nitrate $\delta^{15}N$ profile from GEOTRACES GP16 cruise along ~15 ^{o}S, with the symbols indicating the longitude of DSDP 598 and OC-73-3-20 in the thermocline (19). The GP16 cruise stations are a few hundred kilometers to the north of our study sites. While some GP 16 cruise stations have a little nitrate left at the surface, DSDP 598 and OC-73-3-20 are located well within the oligotrophic gyre with zero nitrate left at the surface. Black lines indicate isopycnals. (**C**) Nitrate sourced from the ODZs as derived from the GP16 nitrate $\delta^{15}N$ and $\delta^{18}O$ data (19), which shows that the thermocline nitrate at our study sites is dominantly sourced from the South Pacific ODZs.

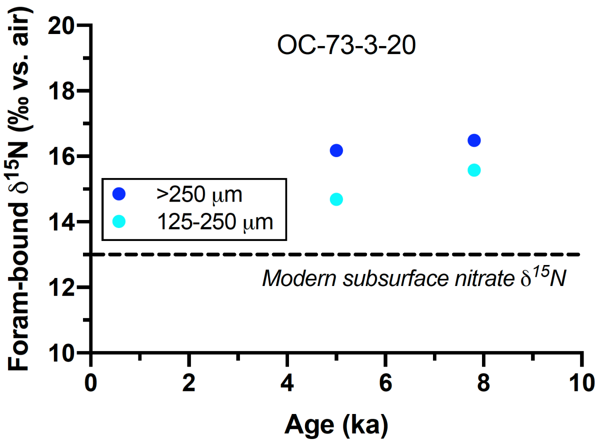

Fig. S2 Holocene foraminifera-bound $\delta^{15}N$ in OC-73-3-20 compared with the $\delta^{15}N$ of the modern subsurface/thermocline nitrate. The high Holocene foraminifera-bound $\delta^{15}N$ values in OC-73-3-20 are consistent with the high modern thermocline nitrate $\delta^{15}N$, indicating the foraminifera-bound $\delta^{15}N$ at the SEPR sites accurately captured the ODZs denitrification signal in the South Pacific.

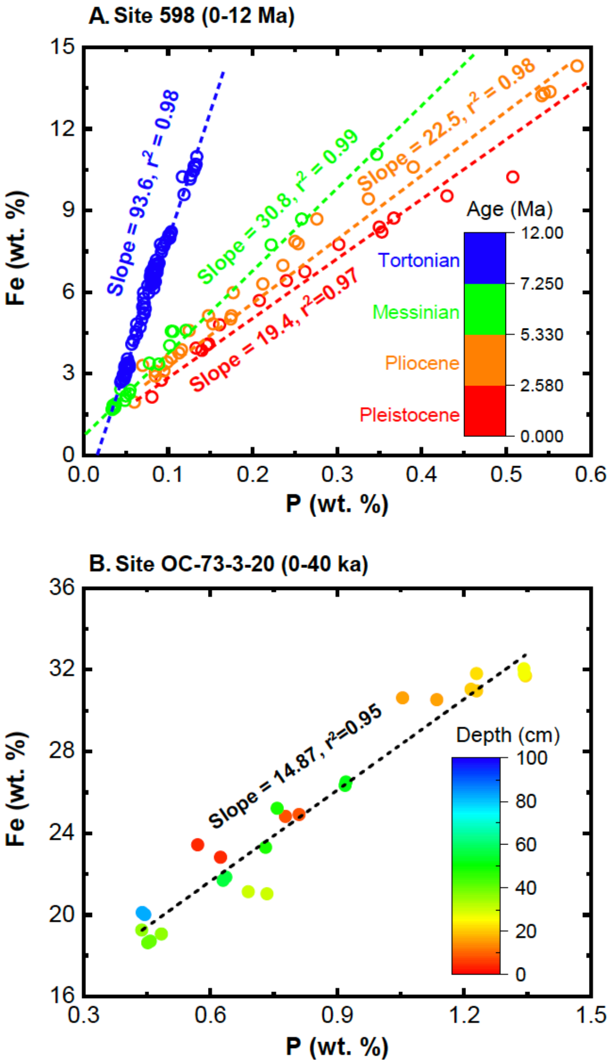

Fig. S3 Cross-plots of Fe (wt. %) and P (wt. %) in (**A**) DSDP 598 since 12 Ma and (**B**) OC-73-3-20 since the last ice age. Fe-P slope in OC-73-3-20 is only slightly higher than the Fe-P slope of Pleistocene samples in DSDP 598 (19.4 ± 1.76 *vs.* 14.87 ± 1.52), indicating the robustness of this relationship. In DSDP 598, during 4 different time intervals (Pleistocene, Pliocene, Messinian, and Tortonian), Fe and P all show strong correlations (r^2>0.97), with different slopes of these cross-plots indicating changing phosphate concentration at the site.

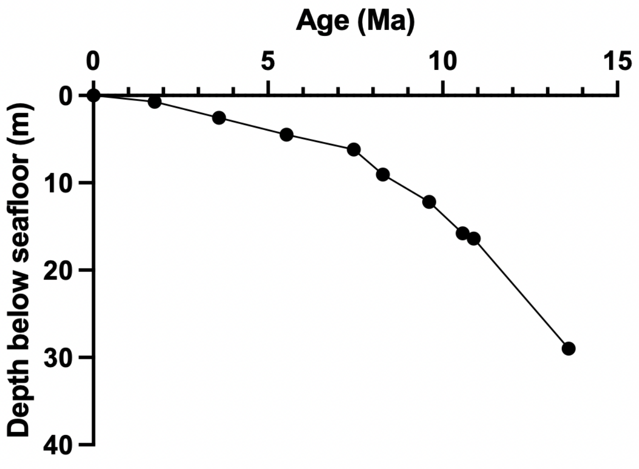

Fig. S4 Updated age model for DSDP 598.

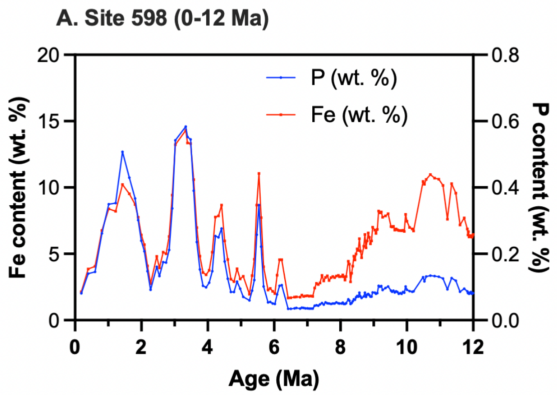

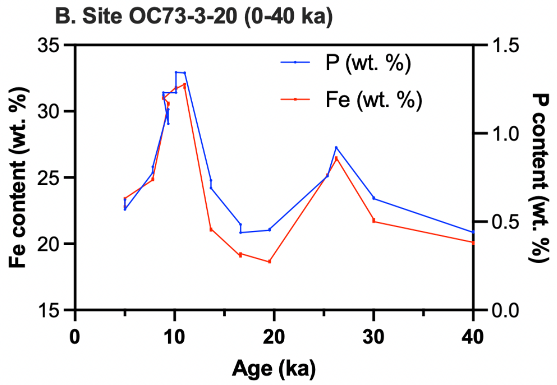

Fig. S5 Timeseries of Fe and P content at DSDP 598 (A) and OC73-3-20 (B).

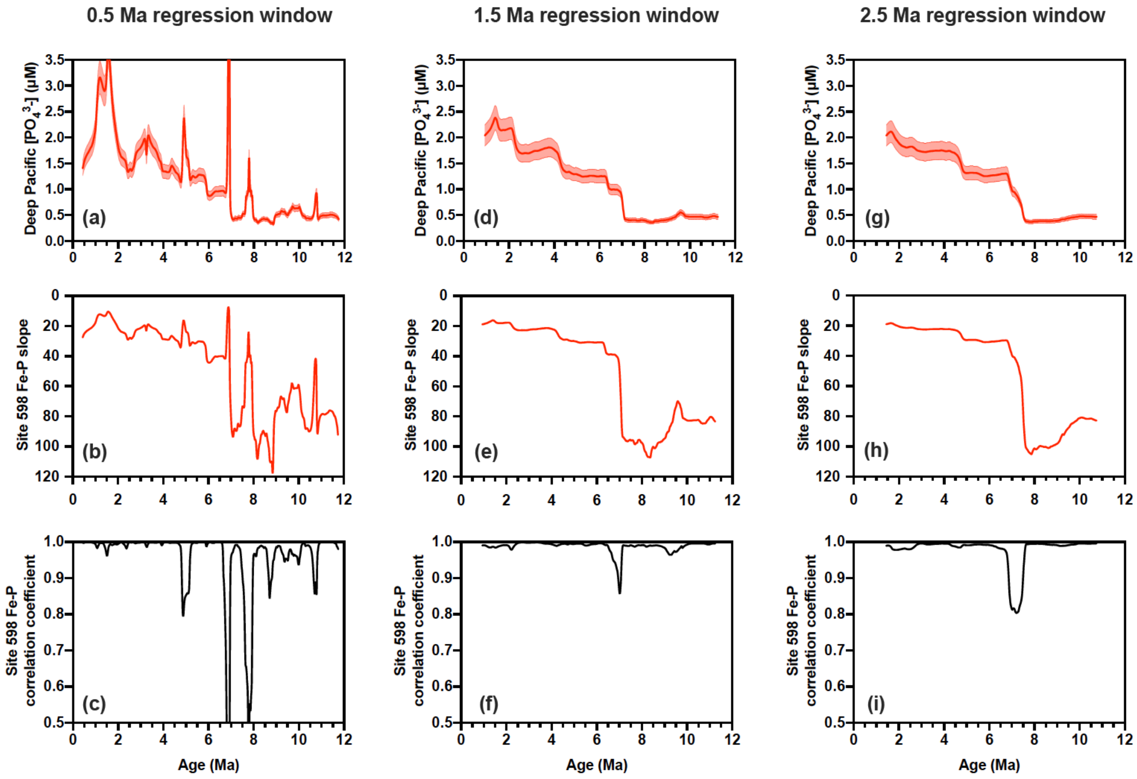

Fig. S6 Running slopes, correlation coefficients, and reconstructed phosphate concentrations using the DSDP 598 Fe and P correlations with a regression window size of 0.5 Ma (a-c),1.5 Ma (d-f), and 2.5 Ma (g-i). The best correlation coefficients were achieved with a regression window size of 1.5 Ma (>0.85). Therefore, we chose a window size of 1.5 Ma for the phosphate reconstructions shown in Fig. 3. The error envelopes were calculated using Monte Carlo approach, accounting for all the uncertainties in the regression.

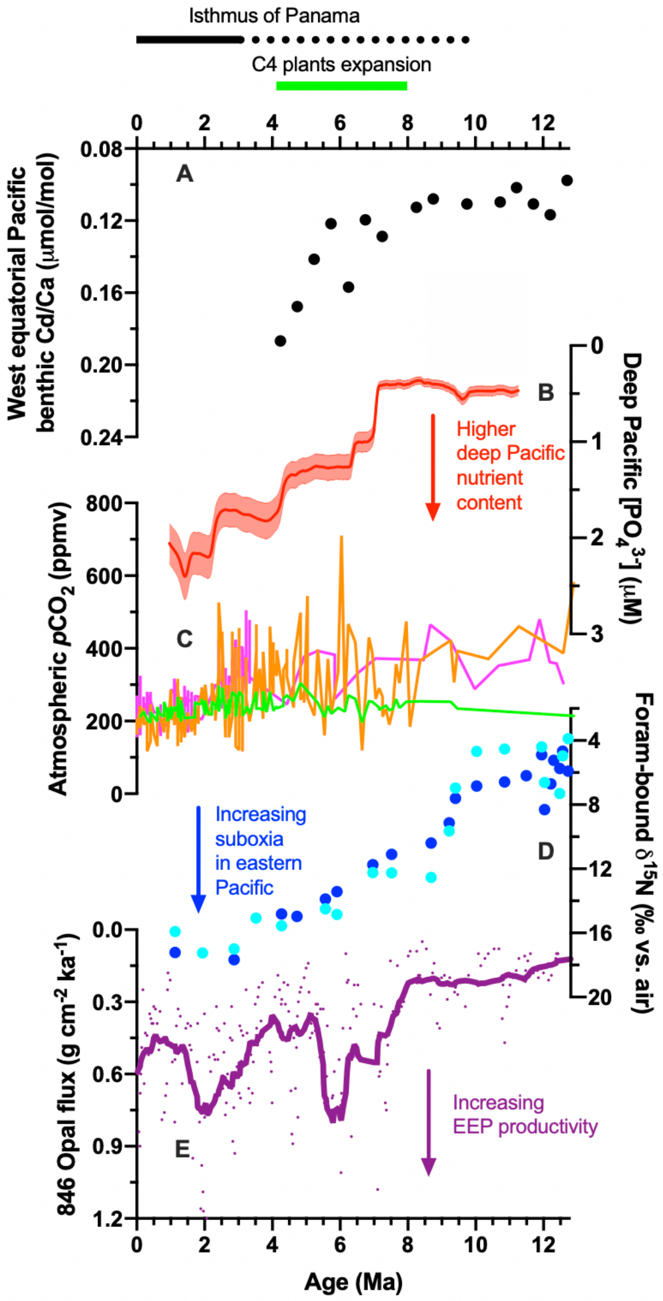

Fig. S7 Comparison of the reconstructed phosphate concentration (**B**) and foraminifera-bound $\delta^{15}N$ (**D**) records from DSDP 598 with: (**A**) A Cd/Ca record from the western equatorial Pacific (20); (**C**) atmospheric *p*CO2 record based on alkenone $\delta^{13}C$ (green and orange lines) and foraminifera $\delta^{11}B$ (pink line) (21); (**E**) Opal flux record (ODP 846) from the eastern Equatorial Pacific (22).

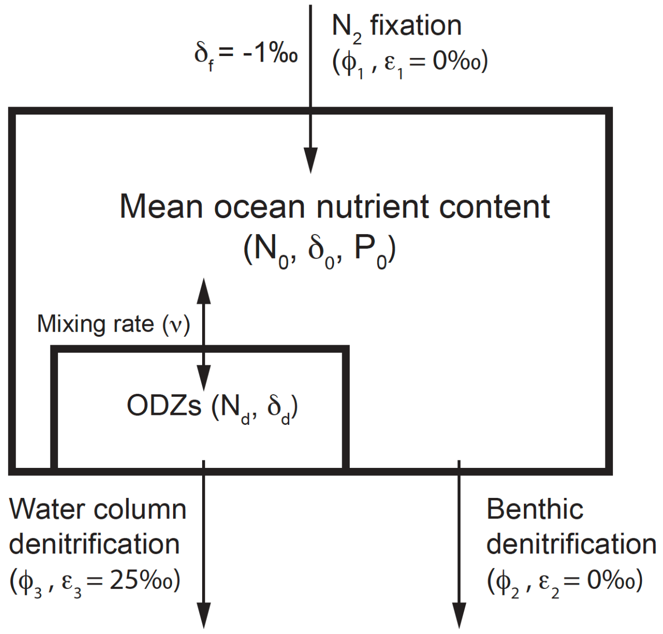

Fig. S8 Framework of the two-box model used to calculate the change of water-column denitrification rate and mean ocean nitrate $\delta^{15}N$ (Fig. 3). In this model, the ODZs box (<1% of total ocean volume) was separated from the rest of the ocean and the communication of the ODZs box with the rest of the ocean ("mean ocean") is described by a mixing term (υ). The input of fixed N to the ocean is through N_2 fixation (ϕ_1) in the "mean ocean" box, which has a fixed $\delta^{15}N$ of -1‰ and no isotopic fractionation. There are two nitrogen loss pathways in this model: water-column denitrification and benthic denitrification. Water-column denitrification is confined to the ODZs box, with an isotope effect of 25‰ (ϕ_3). Benthic denitrification happens in the "mean ocean" box with no isotope effect (ϕ_2). In the "mean ocean" box, the nitrate concentration is assumed to be 16 times of phosphate concentration (Redfield ratio).

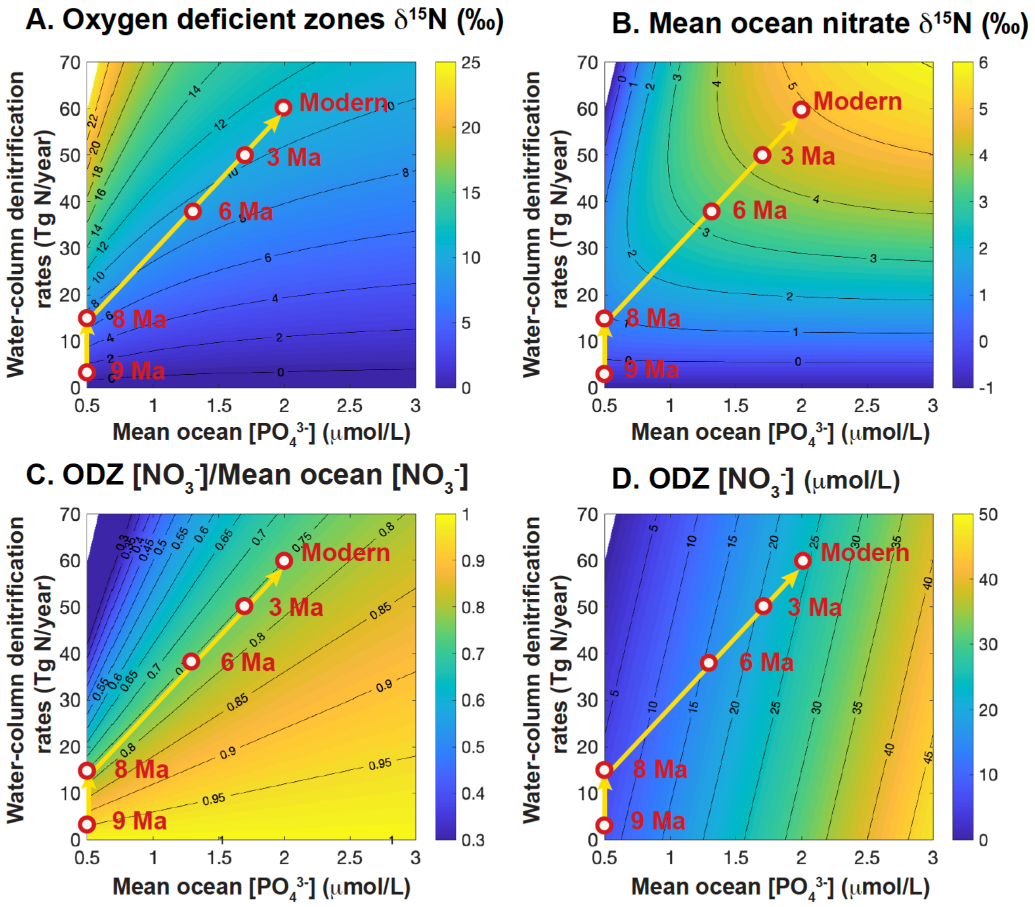

Fig. S9 Model results as in Fig. 2 (**A** and **B**), with two additional panels showing coeval changes in the ratio of ODZs nitrate to mean ocean nitrate (**C**) and ODZs nitrate concentrations (**D**) since 12 Ma.

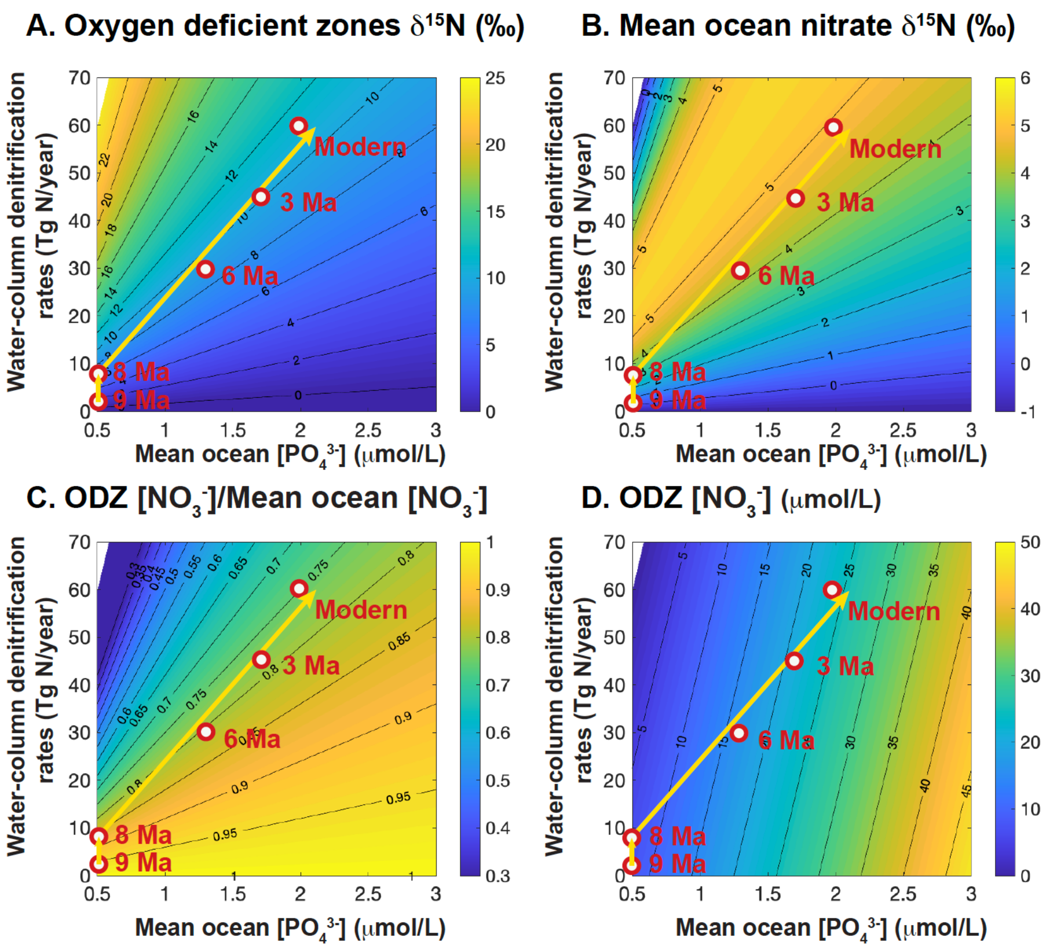

Fig. S10 Model results as in Fig. S6, with benthic denitrification rates scaling with the mean ocean $[PO_4^{3-}]$ ($\phi_2 = [PO_4^{3-}]/[PO_4^{3-}]_{modern}$ x 130 Tg N/year), rather than held constant. Despite this parameter change, water-column denitrification rates still showed a tight correlation with the mean ocean $[PO_4^{3-}]$, with a slightly higher slope than in Fig. 3A (~35 vs. ~31 Tg N/year/μM $[PO_4^{3-}]$).

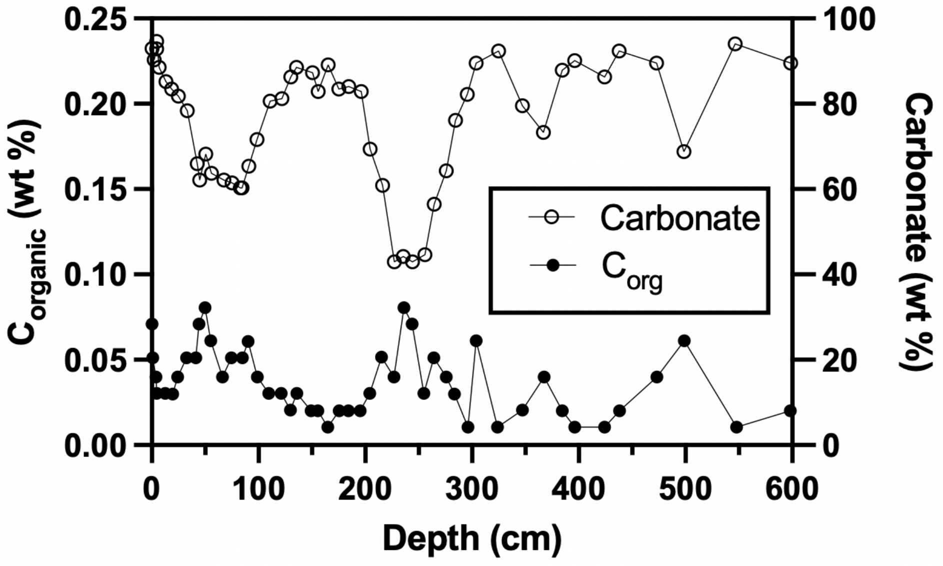

Fig. S11 Organic C and carbonate content in the upper 600 cm of DSDP 598 (7). The organic C content is very low (<0.1%), such that the diagenetic impacts on the P and Fe content are minimal at this site.

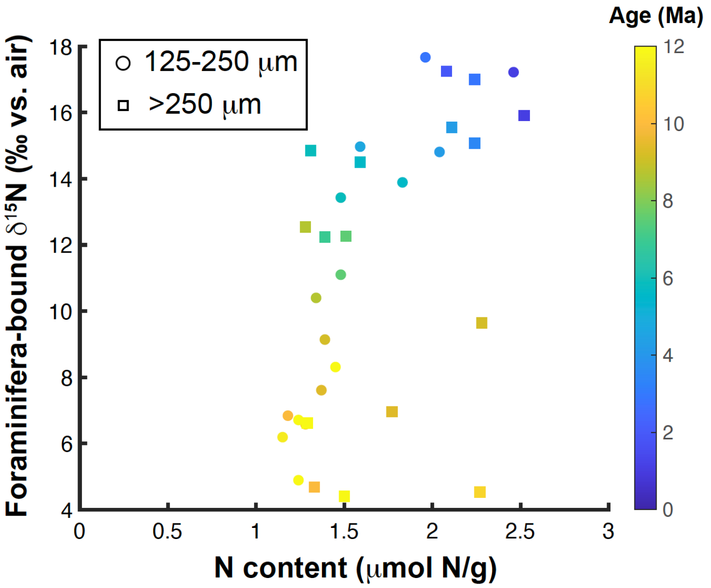

Fig. S12 N content *vs.* $\delta^{15}N$ in DSDP 598 foraminifera with the color indicates the age of the samples. A negative correlation between N content *vs.* $\delta^{15}N$ is generally expected if the $\delta^{15}N$ values have been impacted by diagenesis, because of the isotopic fractionation associated with organic nitrogen breakdown and loss (23). The absence of such negative correlations between N content *vs.* $\delta^{15}N$ in DSDP 598 indicated that diagenesis did not influence the $\delta^{15}N$ values.

SI References

1. K. L. Casciotti, Nitrogen and Oxygen Isotopic Studies of the Marine Nitrogen Cycle. *Annu Rev Mar Sci* 8, 379-407 (2016).

2. D. M. Sigman, *et al.*, The dual isotopes of deep nitrate as a constraint on the cycle and budget of oceanic fixed nitrogen. *Deep Sea Res Part Oceanogr Res Pap* 56, 1419–1439 (2009).

3. H. Ren, D. M. Sigman, R. C. Thunell, M. G. Prokopenko, Nitrogen isotopic composition of planktonic foraminifera from the modern ocean and recent sediments. *Limnol Oceanogr* 57, 1011–1024 (2012).

4. X. T. Wang, *et al.*, Deep-sea coral evidence for lower Southern Ocean surface nitrate concentrations during the last ice age. *Proc National Acad Sci* 114, 3352–3357 (2017).

5. A. S. Studer, *et al.*, Ice age-Holocene similarity of foraminifera-bound nitrogen isotope ratios in the eastern equatorial Pacific. *Paleoceanogr Paleoclimatology* 36, e2020PA00406 (2021).

6. R. A. Berner, Phosphate removal from sea water by adsorption on volcanogenic ferric oxides. *Earth Planet Sc Lett* 18, 77–86 (1973).

7. S. W. Poulton, D. E. Canfield, Co-diagenesis of iron and phosphorus in hydrothermal sediments from the southern East Pacific Rise: Implications for the evaluation of paleoseawater phosphate concentrations. *Geochim Cosmochim Ac* 70, 5883–5898 (2006).

8. E. R. Kast, *et al.*, Nitrogen isotope evidence for expanded ocean suboxia in the early Cenozoic. *Science* 364, 386–389 (2019).

9. S. M. Smart, *et al.*, Ground-truthing the planktic foraminifer-bound nitrogen isotope paleo-proxy in the Sargasso Sea. *Geochim Cosmochim Ac* 235 (2018).

10. H. Ren, *et al.*, Foraminiferal Isotope Evidence of Reduced Nitrogen Fixation in the Ice Age Atlantic Ocean. *Science* 323, 244-248 (2009).

11. R. A. Berner, J. M. VandenBrooks, P. D. Ward, Oxygen and Evolution. *Science* 316, 557–558 (2007).

12. T. D. Herbert, *et al.*, Late Miocene global cooling and the rise of modern ecosystems. *Nat Geosci* 9, 843–847 (2016).

13. J. L. Sarmiento, N. Gruber, *Ocean Biogeochemical Dynamics* (Princeton University Press), (2013).

14. W. Fu, F. Primeau, J. K. Moore, K. Lindsay, J. T. Randerson, Reversal of Increasing Tropical Ocean Hypoxia Trends With Sustained Climate Warming. *Global Biogeochem Cy* 32, 551–564 (2018).

15. T. DeVries, C. Deutsch, P. A. Rafter, F. Primeau, Marine denitrification rates determined from a global 3-D inverse model. *Biogeosciences* 10, 2481–2496 (2013).

16. C. Deutsch, D. M. Sigman, R. C. Thunell, A. N. Meckler, G. H. Haug, Isotopic constraints on glacial/interglacial changes in the oceanic nitrogen budget. *Global Biogeochem Cy* 18, GB4012 (2004).

17. D. Bianchi, J. P. Dunne, J. L. Sarmiento, E. D. Galbraith, Data-based estimates of suboxia, denitrification, and N2O production in the ocean and their sensitivities to dissolved O2. *Global Biogeochem Cy* 26, GB2009 (2012).

18. P. A. Rafter, A. Bagnell, D. Marconi, T. DeVries, Global trends in marine nitrate N isotopes from observations and a neural network-based climatology. *Biogeosciences* 16, 2617–2633 (2019).

19. B. D. Peters, P. J. Lam, K. L. Casciotti, Nitrogen and oxygen isotope measurements of nitrate along the US GEOTRACES Eastern Pacific Zonal Transect (GP16) yield insights into nitrate supply, remineralization, and water mass transport. *Mar Chem* 201, 137–150 (2018).

20. M. L. Delaney, Miocene benthic foraminiferal Cd/Ca records: South Atlantic and western equatorial Pacific. *Paleoceanography* 5, 743–760 (1990).

21. J. W. B. Rae, *et al.*, Atmospheric CO2 over the Past 66 Million Years from Marine Archives. *Annu Rev Earth Pl Sc* 49, 1–33 (2021).

22. J. W. Farrell, *et al.*, Proceedings of the Ocean Drilling Program, 138 Scientific Results. *Proc Ocean Drill Program* (1995).

23. R. S. Robinson, *et al.*, A review of nitrogen isotopic alteration in marine sediments. *Paleoceanography* 27 (2012).
